# Detecting Malaria Hotspots: A Comparison of Rapid Diagnostic Test, Microscopy, and Polymerase Chain Reaction

**DOI:** 10.1093/infdis/jix321

**Published:** 2017-07-07

**Authors:** Polycarp Mogeni, Thomas N Williams, Irene Omedo, Domtila Kimani, Joyce M Ngoi, Jedida Mwacharo, Richard Morter, Christopher Nyundo, Juliana Wambua, George Nyangweso, Melissa Kapulu, Gregory Fegan, Philip Bejon

**Affiliations:** 1 KEMRI-Wellcome Trust Research Programme, Kilifi, Kenya; 2 Faculty of Medicine, Imperial College London; 3 The Jenner Institute, Nuffield Department of Medicine, University of Oxford; 4 Centre for Tropical Medicine and Global Health, Nuffield Department of Clinical Medicine, University of Oxford; 5 Swansea Trials Unit, Swansea University Medical School, Swansea, United Kingdom

**Keywords:** polymerase chain reaction, microscopy, rapid diagnostic test, asymptomatic parasitemia, stable hotspots

## Abstract

**Background:**

Malaria control strategies need to respond to geographical hotspots of transmission. Detection of hotspots depends on the sensitivity of the diagnostic tool used.

**Methods:**

We conducted cross-sectional surveys in 3 sites within Kilifi County, Kenya, that had variable transmission intensities. Rapid diagnostic test (RDT), microscopy, and polymerase chain reaction (PCR) were used to detect asymptomatic parasitemia, and hotspots were detected using the spatial scan statistic.

**Results:**

Eight thousand five hundred eighty-one study participants were surveyed in 3 sites. There were statistically significant malaria hotspots by RDT, microscopy, and PCR for all sites except by microscopy in 1 low transmission site. Pooled data analysis of hotspots by PCR overlapped with hotspots by microscopy at a moderate setting but not at 2 lower transmission settings. However, variations in degree of overlap were noted when data were analyzed by year. Hotspots by RDT were predictive of PCR/microscopy at the moderate setting, but not at the 2 low transmission settings. We observed long-term stability of hotspots by PCR and microscopy but not RDT.

**Conclusion:**

Malaria control programs may consider PCR testing to guide asymptomatic malaria hotspot detection once the prevalence of infection falls.

## Teaser

Detection of hotspots depends on the sensitivity of commonly used diagnostic tools. In the elimination stage, malaria control programs should consider polymerase chain reaction testing to guide detection of asymptomatic malaria hotspots.

**(See the editorial commentary by White on pages 1051–52.)**

The last two decades have witnessed marked declines in *Plasmodium falciparum* malaria transmission in parts of Africa and sustained investment toward malaria control interventions [1, 2]. However, malaria remains a public health challenge in sub–Saharan Africa. Declining transmission intensity is associated with increased microheterogeneity, which complicates effective implementation of malaria control interventions. Mathematical models have shown that targeting control interventions on hotspots would achieve greater impact on reducing malaria transmission intensity than using the same amount of resources for untargeted, blanket coverage [3]. Successful targeting of malaria can only be achieved if hotspots are accurately detected with the currently available diagnostic tools [4].

Cross-sectional surveys that estimate asymptomatic parasite prevalence provide a practical way to assess transmission intensity in the community. However, the estimates of parasite prevalence vary considerably depending on the diagnostic tool, age of study participants, and transmission intensity [5–12]. Rapid diagnostic tests (RDTs), light microscopy, and polymerase chain reaction (PCR) are the diagnostic tools currently being widely used for the assessment of parasite prevalence in the community [13].

Rapid diagnostic tests detect the presence of *P. falciparum* antigens in the blood, either histidine-rich protein 2 (HRP2) or lactate dehydrogenase (pLDH). This tool has greatly improved the ability to provide diagnostic services in rural areas of sub–Saharan Africa because RDTs require minimal training and rely on immune-chromatography, which avoids the need for electricity [14]. Although PCR is a highly sensitive diagnostic tool, it is relatively expensive and requires laboratory support. In comparison with PCR, light microscopy examination of blood smears for malaria parasites (the most commonly used diagnostic tool in clinical and epidemiological studies) has the advantages of lower cost and simplicity but has the disadvantage of limited sensitivity, especially among individuals with submicroscopic infection (ie, parasite densities below microscopy detection limits). Previous studies have shown that malaria parasite densities vary according to the stage of the infection [7], level of acquired immunity [5, 7] and possibly the genetic diversity of circulating parasite clones [15]. Okell et al, in a systematic review and meta-analysis, observed a high proportion of submicroscopic infections (ie, positive by PCR but negative by microscopy or RDT) in low transmission areas and among adults [7]. Therefore, it has been proposed that DNA amplification-based technologies be used to provide adequate sensitivity in the detection of asymptomatic parasitemia cases and hotspots of malaria transmission [5, 7]. However, there are few studies that have examined the extent to which hotspots detected by RDT or microscopy overlap geographically with hotspots detected by PCR using field data.

In a recent study, the efficacy of targeted control interventions was assessed in a cluster randomized controlled trial in Rachuonyo South District in western Kenya [16]. The trial yielded temporary modest declines in malaria transmission both inside and outside the hotspots. On the Kenyan coast, hotspots of asymptomatic parasitemia, as detected by microscopy, were shown to be stable over several years, but hotspots of febrile malaria were not [17]. The stability of asymptomatic parasitemia hotspots presents an opportunity for targeted control, if such hotspots are identified accurately.

The aim of this study is to quantify the extent to which hotspots of malaria transmission detected by RDT and microscopy overlap geographically with those detected by PCR and to examine the variability in temporal stability of hotspots identified by the 3 diagnostic tools. Here we report an analysis of data collected through cross-sectional surveys between 2007 and 2016 from 3 sites experiencing variable transmission intensities within Kilifi County on the Kenyan coast.

## METHODS

### Ethics Statement

Approval for human participation in cross-sectional surveys was given by the Kenya Medical Research Institute Ethics Research Committee. Before any study procedure, written informed consent was obtained from all individuals participating in the surveys, or, where appropriate, guardian/parental consent was sought for children. The studies were conducted according to the principles of the Declaration of Helsinki.

### Study Sites

**Figure 1.**
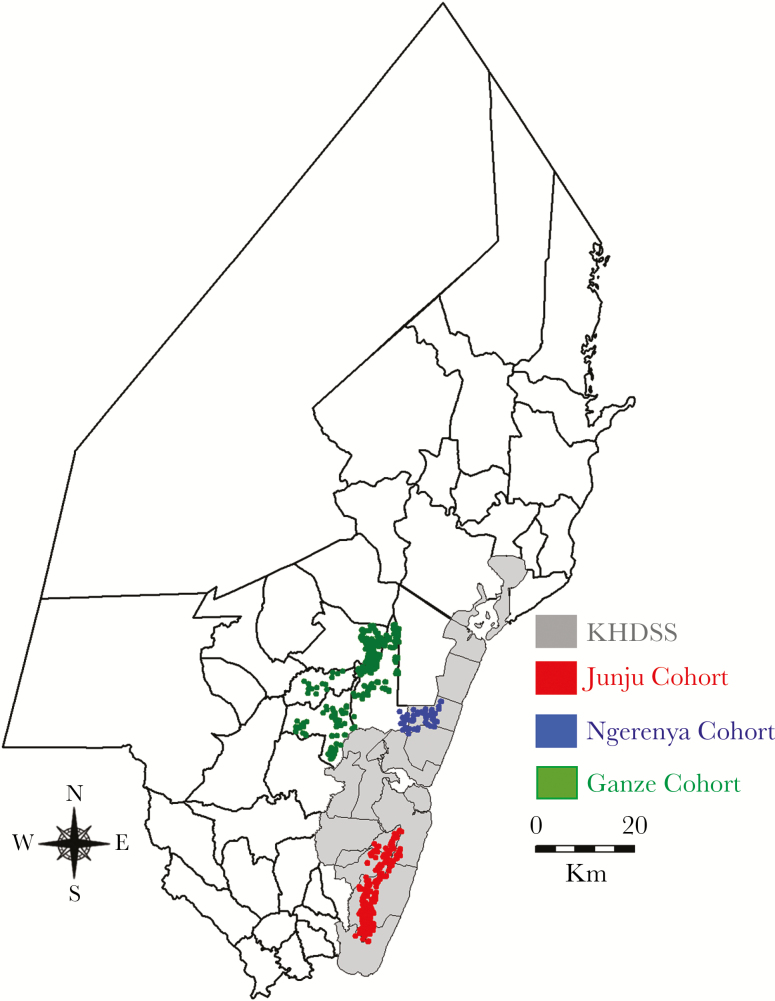
Map of Kilifi County showing the Kilifi Health and Demographic Surveillance System area (shaded gray) and the homesteads where the studies were conducted. Abbreviation: KHDSS, Kilifi Health and Demographic Surveillance System.

We analyzed data from annual cross-sectional surveys conducted within 3 separate cohort studies in Kilifi County on the Kenyan coast. The Junju cohort is located within the southern part of the Kilifi Health and Demographic Surveillance System area (Figure 1) [18] and experiences perennially higher malaria transmission intensity [19] compared with the Ngerenya and Ganze cohorts, which are located to the north. Annual surveillance of asymptomatic malaria in these cohorts is described in detail elsewhere [6, 17]. Briefly, cross-sectional surveys were undertaken annually between 2007 and 2016 in Junju and between 2007 and 2014 in Ngerenya [17]. Surveys took place in April and May of each year, just before the rainy season, and all individuals recruited to the study cohorts were invited to participate by providing a blood sample for malaria diagnosis. In Ganze, 2 cross-sectional surveys were conducted, the first between July and September 2012, and the second between May and July 2013 [6]. Global positioning system coordinates were linked to every homestead in each cohort.

### Field Procedures

Examination for malaria parasites using RDTs, microscopy, and PCR was performed by trained laboratory technicians and was standardized across the sites. Blood samples were obtained from all children aged <15 years whose consent to participate in the study had been obtained [6, 17]. Children with fever (ie, axillary temperature >37.5°C) were referred for immediate assessment and treatment and not included in the survey data. Each sample collected was assessed for parasitemia using RDT, microscopy, and PCR in all sites. Laboratory technologists assessing malaria using any given diagnostic tool were blinded from the result of the other diagnostic tools.

Rapid diagnostic tests (CareStart Malaria Test; AccessBio Inc.) were used to detect the presence of HRP2 specific to *P. falciparum* in the blood. Rapid diagnostic test stocks were stored in air-conditioned rooms with monitored temperature and humidity. Quality assurance for the stored test kits was conducted regularly before use.

Thick and thin blood smears were Giemsa stained and examined using light microscopy at 1000× magnification for malaria parasites and malaria species, respectively. Malaria infection and parasite counts by microscopy were determined independently by 2 readers, and discordant readings were resolved by a 3rd reader. The number of parasites per 200 white blood cells (WBCs) was counted, and parasite density per microliter of blood was calculated using an average count of 8000 WBCs/µL of blood, as described elsewhere [20], and reported by species (ie, *P. falciparum*, *Plasmodium malariae,* and *Plasmodium ovale*).

For PCR analysis, DNA was first extracted from 30 µL of whole blood using QIAxtractor machine (QIAGEN, Hilden, Germany). The DNA was eluted in 100 µL, from which 5 µL of DNA were amplified by quantitative PCR. This was done using a TaqMan assay for the *P. falciparum* multicopy 18S ribosomal RNA genes, as described elsewhere [21], except we used a modified probe (5′-FAM-AACAATTGGAGGGCAAG-NFQ-MGB-3′), as described elsewhere [22]. We used an Applied Biosystems 7500 Real-Time PCR System with quantification by Applied Biosystems 7500 software v2.0.6. Samples were analyzed in singlet wells. Three negative control wells and 7 serial dilutions of DNA extracted from in vitro parasite cultures were included as standards on each plate in triplicate [23]. Plates failing quality control standards were repeated. The lower limit of accurate quantification of this method is 10 parasites/mL within the PCR elute, and by assessing 1/20 of 30 µL of blood with a gene target present on 3 chromosomes. The method has a theoretical limitation of 4.5 parasites/µL of whole blood, compared with a sensitivity of 50 parasites/µL for thick blood films. Rapid diagnostic test, microscopy, and PCR standards were monitored through a quality assurance scheme that included comprehensive training during induction and at regular intervals during the study period. Microscopy quality assurance was evaluated using external quality control slides.

### Geographical Cluster Analysis

Individuals who had complete data on RDT, PCR, and microscopy were included in the analysis. Hotspots are defined as geographical areas experiencing significantly higher prevalence of asymptomatic parasitemia than would be expected by chance. In our study, we assess chance using the spatial scan statistic [24] through the Bernoulli model in SaTScan software v9.4.1. This software imposes a scanning window (set to “circular” in this analysis) that moves systematically across geographical space with radius varying from zero to a maximum radius enclosing a prespecified population size (at most 30% in this analysis) in the sampling frame. For each location and size of the window, the number of observed cases are counted, and expected cases are computed by assuming a uniform distribution of cases across the population. The scan statistic compared the count within each circle with that outside to derive a log likelihood statistic. To test the null hypothesis of complete spatial randomness, a Monte-Carlo simulation was used to generate permutations of the observed cases across the entire set of data locations, and the observed log likelihood was compared with the simulated log likelihoods to determine significance [24]. Local clusters of RDT, PCR, and microscopy data were assessed separately, and the differences in parameters (ie, risk ratios [RR], hotspots radius, and *P*- alues) were compared. The risk ratio herein is defined as the risk of malaria within a hotspot divided by the risk outside the hotspot.

### Temporal Variation in Malaria Transmission

Parasite prevalence was computed by imposing spatial grids on the data and collapsing to the mean prevalence within each cell of the grid. This was done with grids of variable sizes—0.5 × 0.5 km, 1 × 1 km, and 2 × 2 km—selected a priori to allow for a sensitivity analysis that would examine the potential bias resulting from the modifiable areal unit problem and repeated by year. The association between parasite prevalence by PCR and by microscopy or RDT was assessed for the various grid sizes. Furthermore, we compared the stability of spatial heterogeneity of PCR and microscopy datasets by examining Spearman’s rank correlation coefficient between parasite prevalences within grids separated in time.

The degree to which hotspots overlap was defined as the fraction of homesteads within the intersection of hotspots detected by PCR and microscopy or RDT divided by the total number of homesteads within the hotspots. Only homesteads within primary hotspots (most likely cluster regardless of significance) and any other significant secondary clusters were included in the computations.

Hotspots of malaria transmission were mapped on Google Map extracts in R version 3.3.1 [25]. Graphs, Kappa statistics, and correlation analyses were done using Stata version 12.

## RESULTS

**Figure 2.**
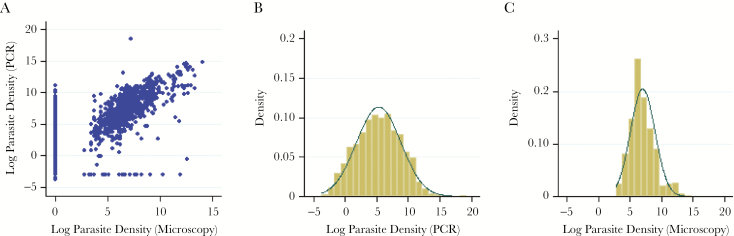
Distribution of parasite densities. *A*, Scatter plot of log-transformed parasite per microliter densities detected by microscopy and polymerase chain reaction (PCR). Polymerase chain reaction–negative test results were assigned an arbitrary value of 0.05 parasite/µL, whereas microscopy-negative test results were assigned an arbitrary value of 1 parasite/µL before log transformation to allow complete data presentation for samples that were positive by either PCR or microscopy. *B* and *C*, Histograms of log-transformed PCR and microscopy parasites per microliter densities, respectively, against normal distribution functions. Abbreviation: PCR, polymerase chain reaction.

A total of 8581 study participants were surveyed in the 3 study sites. There was a positive correlation between *P. falciparum* parasite density measured by PCR and by microscopy among those testing positive (Figure 2A) (*r* = 0.72; *P* < .001) and strong association between detection by PCR and detection by microscopy (Supplementary Table 1) (kappa = 0.6159; *P* < .001). Parasite densities by PCR and microscopy were log-normally distributed (Figure 2B and 2B). The geometric mean PCR densities (of positive samples) were lowest in Ngerenya (11.79 parasites/µL; 95% confidence interval [CI] = 3.68–37.76 parasites/µL) and highest in Junju (220.02 parasites/µL; 95% CI = 184.17–262.85 parasites/µL).

### Hotspots of Malaria Transmission

Malaria species were only examined by microscopy. Overall, the prevalences of malaria by species in the 3 sites were 9.67% (n = 830/8581 films), 0.16%(n = 13/8004 films), 0.60% (n = 48/8014 films), and 0% (n = 0/8014 films) for *P. falciparum*, *P. ovale, P. malariae* and *Plasmodium vivax* respectively. *Plasmodium ovale* and *P. malariae* were only detected in the moderate transmission site (Junju) and not in either of the low transmission sites. No *P. vivax* case was reported in any of the sites.

In pooled data analysis from Junju, we identified 2 statistically significant hotspots of *P. falciparum* (radius = 1.75 km; RR = 2.69; *P* < .001) and (radius = 1.07 km; RR = 2.87; *P* < .001). We identified 1 significant primary hotspot of *P. malariae* (radius = 0.053 km; RR = 10.41; *P* = .003), a borderline significant secondary hotspot (radius = 0 km; RR = 9.33; *P* = .07), and a nonsignificant hotspot of *P. ovale* (radius = 1.76 km; RR = 6.3; *P* = .44). The hotspots of *P. falciparum*, *P. malariae*, and *P. ovale* overlapped geographically (supplementary figure 1). Further analysis was restricted to *P. falciparum.*

**Figure 3.**
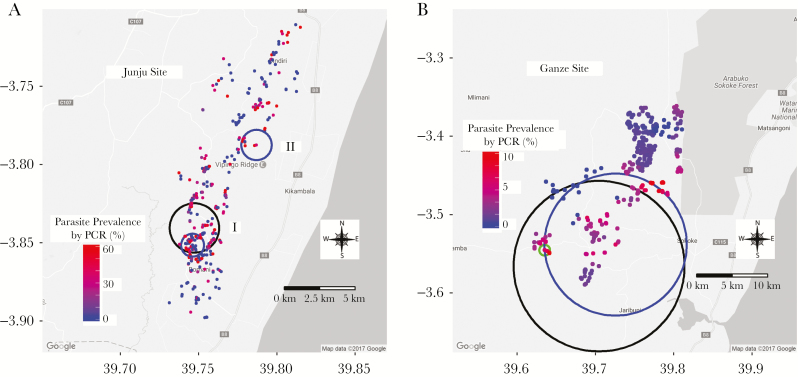
Hotspots of malaria transmission. *B*, Junju cohort. *B*, Ganze cohort. In Junju, there was complete overlap between polymerase chain reaction (PCR; black circles) and microscopy (green circles) but partial overlap by rapid diagnostic test (RDT; blue) for the primary hotspot (I). However, for the 3 diagnostic tools used, there was complete overlap in the significant secondary hotspots (II). In Ganze, the hotspot detected by microscopy (green circle) was within the hotspots detected by PCR (black circle) and at the border with RDT (blue circle). Abbreviation: PCR, polymerase chain reaction.

*Plasmodium falciparum* was detected by PCR, RDT, and microscopy. Significant hotspots of malaria transmission by the 3 diagnostic tools were observed in the Junju and Ganze sites. However, hotspots of malaria transmission in Ngerenya were statistically significant only when measured by PCR and RDT and not statistically significant when measured by microscopy (Table 1). Overall (pooled data analysis across all years of monitoring), the degree of overlap between hotspots detected by PCR and those detected by microscopy was 100% in Junju, but less overlap was noted when hotspots were examined year by year (Table 1 and Figure 3). However, in the Junju site, there was partial overlap of primary hotspots detected by PCR and RDTs (45.9%) but complete overlap for the significant secondary hotspots (Table 1). Overall, overlaps in hotspots detected in Ganze and Ngerenya sites were inconsistent. The risk ratios for microscopy hotspots were consistently larger than those measured by PCR.

**Table 1.** Properties of Malaria Hotspots and Degree of Homestead Overlap Between Hotspots Detected By Polymerase Chain Reaction, Microscopy, and Rapid Diagnostic Test

Study		PCR			Microscopy			RDT			Degree of overlap (%)		
	Period	Radius	RR	*P* value	Radius	RR	*P* value	Radius	RR	*P* value	PCR vs microscopy	PCR vs RDT	RDT vs microscopy
Junju	Overall	1.75	1.85	<.001	1.75	2.69	<.001	0.81	1.91	<.001	100	45.9	45.9
	Overall^a^	1.07	2.23	<.001	1.07	2.87	<.001	1.07	2.61	<.001	100	100	100
	2007	0.9	2.17	.003	2.11	4.57	<.001	2.38	4.99	<.001	42.02	39.32	82.22
	2008	1.67	2.2	<.001	1.76	2.22	.002	1.58	3.92	<.001	71.54	72.5	85.71
	2009	2.38	2.34	<.001	1.54	3.4	<.001	1.71	7.88	<.001	68.79	55.84	73.64
	2010	1.77	2.01	<.001	1.78	2.65	<.001	1.97	3.76	<.001	79.2	73.13	63.19
	2011	1.44	2.71	<.001	1.72	6.28	<.001	0.64	5.5	.009	76.42	20.18	23.68
	2012	2.08	2.02	<.001	1.29	2.7	<.001	1.78	2.08	.001	57.63	61.81	57.76
	2013	0.36	3.31	.002	0.19	9.07	.13	1.98	2.69	.001	8.33	8.53	2.33
	2014	0	3.37	.09	0.94	4.11	.10	0.19	2.11	.02	0	0	0
	2015	0.74	2.25	<.001	0.63	3.31	<.001	0.52	2.44	<.001	25.42	48.65	22.64
	2015^a^	0.33	2.68	.02	0.14	3.56	.02	0.33	2.49	.03	25.42	100	0
	2016	0	3.74	.21	0.64	3.19	.12	0.02	3.61	.02	0	0	0
Ganze	Overall	12.12	4.14	<.001	0.76	31	.003	10.08	67.4	<.001	2.75	75.61	2.8
Ngerenya	Overall	1.04	5.35	.005	0	36.6	.12	0	33.8	.02	50	50	100
	2007–2010	0	8.96	.21	1.65	8.11	.76	0	60.69	.06	0	100	0
	2010–2014	0.56	5.2	.02	…	…	…	0.83	14.28	.34	…	25	…

Abbreviations: PCR, polymerase chain reaction; RDT, rapid diagnostic test; RR, relative risk.

^a^Shows significant secondary clusters.

### Association of Parasite Prevalence By Polymerase Chain Reaction, Microscopy, and Rapid Diagnostic Test

In all sites and across all 3 grid sizes examined, there was a strong positive correlation between prevalence of parasitemia measured by PCR and prevalence of parasitemia measured by microscopy or RDT (ie, geographical areas experiencing high malaria prevalence as measured by PCR were also more likely to be high when measured by microscopy or RDT). However, the associations were weaker in low transmission settings (Table 2 and Supplementary Table 2).

**Table 2.** Association Between Parasite Prevalence by Polymerase Chain Reaction and Parasite Prevalence by Microscopy at Various Grid Sizes.

Site	Year	Parasite prevalence		0.5 × 0.5 km grid		1 × 1 km grid		2 × 2 km grid	
		PCR (%)	Microscopy (%)	Correlation (CI)	*P* value	Correlation (CI)	*P* value	Correlation (CI)	*P* value
Junju cohort	Overall	30.10	16.54	0.73 (.70–.76)	<.001	0.81 (.77–.84)	<.001	0.86 (.82–.89)	<.001
	2007	29.82	16.27	0.70 (.50–.83)	<.001	0.70 (.37–.88)	<.001	0.83 (.38–.96)	.005
	2008	47.51	29.33	0.79 (.63–.89)	<.001	0.83 (.62–.94)	<.001	0.93 (.71–.99)	<.001
	2009	31.45	21.36	0.58 (.32–.76)	<.001	0.82 (.58–.93)	<.001	0.90 (.58–.98)	<.001
	2010	39.32	21.98	0.78 (.71–.84)	<.001	0.76 (.63–.85)	<.001	0.83 (.65–.92)	<.001
	2011	26.93	15.48	0.69 (.58–.77)	<.001	0.80 (.69–.87)	<.001	0.88 (.75–.95)	<.001
	2012	27.68	15.40	0.72 (.62–.79)	<.001	0.79 (.67–.87)	<.001	0.80 (.59–.91)	<.001
	2013	19.42	7.89	0.69 (.59–.77)	<.001	0.79 (.67 -.87)	<.001	0.85 (.68–.93)	<.001
	2014	30.32	14.76	0.73 (.64–.81)	<.001	0.83 (.73–.89)	<.001	0.91 (.81–.96)	<.001
	2015	30.75	17.65	0.77 (.61–.88)	<.001	0.76 (.49–.90)	<.001	0.81 (.37–.95)	.005
	2016	23.51	11.26	0.46 (.17–.69)	.004	0.48 (.04–.77)	.04	0.47 (−.22 to .85)	.17
Ngerenya cohort	Overall	2.04	0.21	0.37 (.27–.46)	<.001	0.38 (.26–.48)	<.001	0.40 (.22–.56)	<.001
Ganze cohort	Overall	5.85	1.03	0.45 (.34–.55)	<.001	0.45 (.30–.58)	<.001	0.48 (.28–.63)	<.001
	2012	7.73	1.81	0.51 (.37–.63)	<.001	0.53 (.35–.68)	<.001	0.60 (.34–.77)	<.001
	2013	4.11	0.30	0.35 (.17–.51)	<.001	0.30 (.05–.52)	.02	0.23 (−.11 to .53)	.19

Abbreviatons: CI, confidence interval; PCR, polymerase chain reaction.

### Temporal Stability of Malaria Transmission in the Study Sites

In the Junju site, the prevalences of parasitemia within grids were predictive of the prevalences in the following year. The stability appeared to be greater for PCR and microscopy, which remained significant for intervals <5 years, and less stable for RDT prevalences, which were significantly predictive of the prevalence in the following year for intervals only up to 2 years.

In contrast, the prevalences of parasitemia within grids in Ganze were not predictive for the following year by any measure (Table 3, Supplementary Table 3, and Supplementary Table 4), and we were not sufficiently powered to conduct such analysis in Ngerenya. The findings for temporal stability were consistent across the 3 spatial scales used (0.5 × 0.5 km, 1 × 1 km, and 2 × 2 km).

**Table 3.** Association Between Distribution of Malaria Parasite Prevalence Detected by Microscopy, Polymerase Chain Reaction, and Rapid Diagnostic Test Within 2 × 2 Kilometer Grid Size Over Iime Intervals

Study site	Interval between cluster, y	2 × 2 km grid					
		Microscopy analysis		PCR analysis		RDT analysis	
		Correlation (95% CI)	*P* value	Correlation (95% CI)	*P* value	Correlation (95% CI)	*P* value
Junju cohort	1	0.46 (.32–.58)	<.001	0.41 (.26–.53)	<.001	0.43 (.29–.56)	<.001
	2	0.55 (.41–.66)	<.001	0.44 (.29–.58)	<.001	0.50 (.35–.62)	<.001
	3	0.44 (.26–.59)	<.001	0.34 (.15–.51)	<.001	0.18 (−.02 to .37)	.08
	4	0.46 (.25–.63)	<.001	0.48 (.28–.64)	<.001	0.08 (−.16 to .31)	.53
	5	0.53 (.29–.71)	<.001	0.34 (.06–.57)	.02	0.11 (−.19 to .38)	.48
	6	0.47 (.18–.69)	.003	0.32 (−.01 to .59)	.051	0.27 (−.07 to .55)	.12
	7	0.48 (.12–.73)	.0111	0.65 (.35–.82)	<.001	0.22 (−.17 to .56)	.27
	8	0.33 (−.16 to .69)	.1788	0.27 (−.22 to .66)	.27	0.34 (−.15 to .70)	.17
	9	0.54 (−.19 to .89)	.1318	0.74 (.14–.94)	.02	0.34 (−.42 to .82)	.37
Ganze cohort	1	0.35 (−.08 to .67)	.1075	0.30 (−.14 to .64)	.17	…	…

Similar trends were observed at grid size 0.5 × 0.5 km (Supplementary Table 3) and 1 × 1 km (Supplementary Table 4).

Abbreviations: CI, confidence interval; PCR, polymerase chain reaction; RDT, rapid diagnostic test.

## DISCUSSION

*Plasmodium falciparum* parasite prevalence has frequently been used as a marker of transmission intensity and is widely used in detection of hotspots of asymptomatic parasitemia. However, the estimated prevalence of parasitemia has been shown to vary substantially with the diagnostic tool used. Polymerase chain reaction and other molecular techniques are significantly more sensitive than microscopy and RDT for detection of malaria parasites, especially at lower transmission intensities where parasite densities are lower [7, 8]. This study examines the microepidemiology of malaria transmission in 3 sites on the Kenyan coast that experience varying transmission intensities.

We observed substantial heterogeneity of malaria transmission in the 3 sites, as has been previously described [17]. Hotspots were detected by PCR, RDT, and microscopy and were statistically significant for all sites except by microscopy in the Ngerenya site. When all years from the Junju site were pooled for spatial analysis, hotspots by PCR completely overlapped with hotspots by microscopy and partially overlapped with RDT. However, an analysis of individual year-by-year data showed some variation in the degree of overlap (Table 1). Overlap became less marked in later years, coinciding with reductions in transmission intensity [26], and little overlap was noted in Ganze, where transmission is lower [6]. It is unsurprising that hotspots of the different malaria species overlapped geographically because the different species are transmitted by similar vectors.

There were significant correlations between PCR and microscopy and between PCR and RDT parasite prevalences within grid cells imposed on the data at 3 different spatial scales. The correlations were stronger in Junju than in Ngerenya and Ganze (Table 2). The prevalence of infection was <2% in Ngerenya and Ganze. Taking the findings on degree of overlap of hotspots in the different transmission settings and the correlation between parasite prevalence together, we conclude that hotspots detected by PCR are likely to occur in the same geographical areas as those detected by microscopy at moderate transmission intensities. However, the accuracy with which they overlap is lessened when transmission is less intense.

As would be expected, PCR densities were lower than microscopy densities [27], and the average densities by PCR were lower in low transmission settings (Ngerenya and Ganze) compared with the moderate transmission setting (Junju). Moreover, the proportion of PCR-positive cases that were positive by microscopy were highest in Junju, followed by Ganze and Ngerenya in that order (Supplementary Table 1). Our findings suggest that microscopy and RDT miss a larger proportion of infections in low transmission areas (Ngerenya and Ganze) compared with moderate transmission settings (Junju), which may explain why PCR becomes more important in detecting hotspots at lower transmission intensities.

We observed stable hotspots of asymptomatic parasitemia in the Junju cohort but not in Ganze, and we were not powered to assess stability of hotspots in Ngerenya. Hotspots were similarly stable when detected by PCR or microscopy but not RDT (Table 3). The advent of HRP2-dependent RDTs greatly expanded access to malaria diagnostics tools because of low cost and ease of applicability in the field, but the sensitivity of this technique is lower than that for PCR and may be comparable with the sensitivity of routine microscopy [14]. In addition, the HRP2 antigen can circulate in blood for weeks after treatment, leading to false positives, and recent studies show that some *P. falciparum* parasites do not express the HRP2 protein, leading to false negatives [28]. These factors potentially result in poorer discrimination for the location of hotspots, explaining the lack of long-term stability of hotspots detected by RDT. Furthermore, hotspots defined by RDT did not consistently overlap the PCR or microscopy hotspots. We conclude that although RDTs have a firmly established place in diagnosis of acute fever and malaria indicator surveys [14, 29], their utility for fine-scale mapping of hotspots is less clear.

The main limitation of our study is that data were collected from geographical areas of close proximity on the Kenyan coast. However, these geographical areas captured a range of transmission intensities during a period when transmission was falling [19]. Although the Ngerenya dataset (ie, data from a site with low transmission intensity) was large (n = 2286), there were few positive cases (Supplementary Table 1) and hence limited power to describe and compare hotspots.

Clinical malaria case monitoring has also been used to identify hotspots of malaria transmission [6]. However, this may be less sensitive in identifying stable hotspots of malaria where substantial immunity in the population offsets the risk of clinical malaria [17], and even at low transmission intensity, hotspots determined by PCR do not overlap with microscopy hotspots [6]. Hence PCR monitoring of asymptomatic infection may identify hotspots that would not be detected by monitoring clinical cases and may be useful in pre-elimination surveillance.

### Implications of the Findings

Malaria control programs increasingly need to adopt targeted malaria control at low transmission intensities. Our findings suggest that PCR, RDT, and microscopy can potentially determine hotspots at moderate transmission intensities, but PCR testing has a diagnostic advantage as transmission intensity falls. Therefore, malaria control programs should consider PCR testing when the prevalence of infection is low.

## Supplementary Data

Supplementary materials are available at *The Journal of Infectious Diseases* online. Consisting of data provided by the authors to benefit the reader, the posted materials are not copyedited and are the sole responsibility of the authors, so questions or comments should be addressed to the corresponding author.

## Supplementary Material

supplementary figure 1

supplementary figure legends

Supplementary Tables

## Notes

***Acknowledgements.*** We thank the study participants, microscopy and laboratory personnel, field workers, demographic surveillance personnel, and data managers at the Kemri-Wellcome Trust Research Programme Kilifi. The study is published with the permission of the Director of Kemri. Data that support the findings of this manuscript are available from the Kemri Institutional Data Access/Ethics Committee, for researchers who meet the criteria for access to confidential data. Data are from the annual cross-sectional surveys conducted within the longitudinal cohorts of children that are under routine surveillance for malaria. Details of the criteria can be found in the KEMRI-Wellcome data sharing guidelines (https://kemri-wellcome.org/about-us/#ChildVerticalTab_15). The data includes homestead-level coordinates as an essential component, and these are personally identifiable data. Access to data is provided via the KEMRI-Wellcome Data Governance Committee: Data_Governance_Committee@kemriwellcome.org; +254708 587 210; Contact person, Marianne Munene (Secretary; +254709 983 436). P. B. and P. M. conceived and designed the experiments. D. K., J. M. N., J. M., R. M., J. W., G. N., and M. K. performed the experiments. P. M., T. N. W., I. O., G. F., and P. B. analyzed the data. P. M. wrote the first draft of the manuscript. P. M., T. N. W., I. O., G. F., and P. B. contributed to the writing of the manuscript. P. M., T. N. W., I. O., D. K., J. M. N., J. M., R. M. , J. W., G. N., M. K., G. F., and P. B. agree to the manuscript’s results and conclusions. All authors have read, and confirm that they meet, ICMJE criteria for authorship.

***Financial support.*** This study is funded by the Wellcome Trust (core grants 081829, 079080, 103602). T. N. W. is funded by the Wellcome Trust (grant 091758). P. M., I. O., and P. J. are funded by the UK Medical Research Council (MRC) and the UK Department for International Development (DFID) under the MRC/DFID Concordat agreement (G1002624). R. M. is funded by the Wellcome Trust PhD Studentship (grant 109026/Z/15/Z).

***Potential conflicts of interest.*** All authors: No reported conflicts of interest. All authors have submitted the ICMJE Form for Disclosure of Potential Conflicts of Interest. Conflicts that the editors consider relevant to the content of the manuscript have been disclosed.
