# Supplementary figures and images for: Detecting Malaria Hotspots: A Comparison of Rapid Diagnostic Test, Microscopy, and Polymerase Chain Reaction
Source: J Infect Dis. 2017 Jul 7;216(9):1091–8. doi: 10.1093/infdis/jix321 (PMC5853881; doi:10.1093/infdis/jix321)

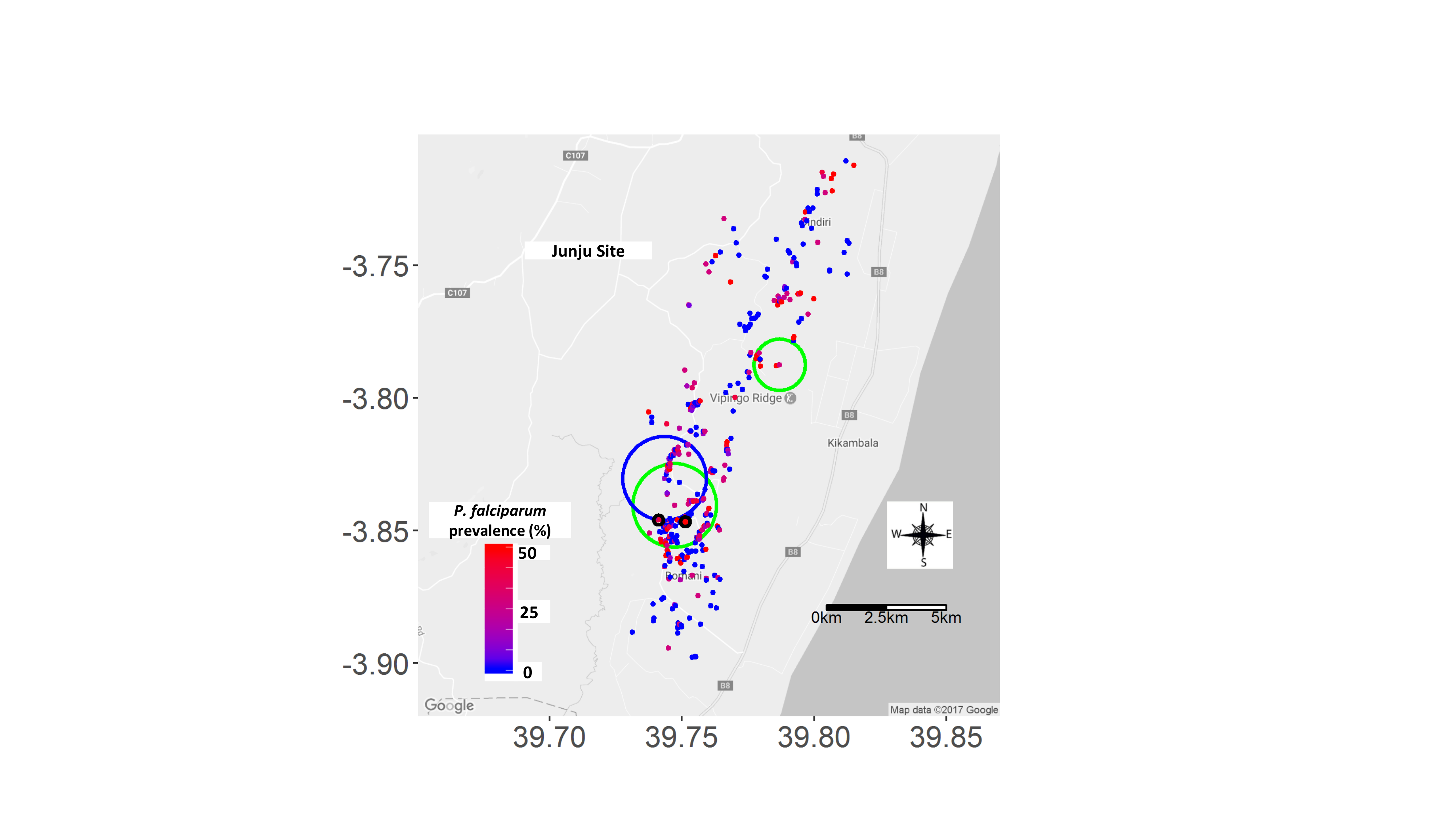

Supplement: supplementary figure 1 [file jix321_suppl_supplementary_fig_1.png]
