# Supplementary material for: Detecting Malaria Hotspots: A Comparison of Rapid Diagnostic Test, Microscopy, and Polymerase Chain Reaction
Source: J Infect Dis. 2017 Jul 7;216(9):1091–8. doi: 10.1093/infdis/jix321 (PMC5853881; doi:10.1093/infdis/jix321)
Supplement: supplementary figure legends [file jix321_suppl_supplementary_figure_legends.docx]

**Supplementary Figure legends**

**Supplementary Fig 1**: Hotspots of malaria transmission by species. Green (significant primary and secondary hotspots), blue (primary non-significant hotspot) and the black (significant primary and secondary hotspots) circles represent hotspots of *P. falciparum*, *P. ovale* and *P. malariae* respectively as detected by microscopy.

**Supplementary Table legends**

**Supplementary Table 1**. Number of samples that were malaria parasite positive by microscopy and PCR

**Supplementary Table 2.** Association between parasite prevalence by PCR and by RDT at various grid sizes

**Supplementary Table 3**. Association between distribution of malaria parasite prevalence detected by microscopy, PCR and RDT within 0.5x0.5 km^2^ grid size over time intervals.

**Supplementary Table 4.** Association between distribution of malaria parasite prevalence detected by microscopy, PCR and RDT within 1x1 km^2^ grid size over time intervals.
