# Supplementary material for: Detecting Malaria Hotspots: A Comparison of Rapid Diagnostic Test, Microscopy, and Polymerase Chain Reaction
Source: J Infect Dis. 2017 Jul 7;216(9):1091–8. doi: 10.1093/infdis/jix321 (PMC5853881; doi:10.1093/infdis/jix321)
Supplement: Supplementary Tables [file jix321_suppl_supplementary_tables.docx]

**Supplementary tables**

**Supplementary Table 1**. Number of samples that were malaria parasite positive by microscopy and PCR

| Site | N | Malaria test result | | |  | |
| --- | --- | --- | --- | --- | --- | --- |
|  |  | Both PCR and Microscopy n(%) | PCR only n(%) | Microscopy only n(%) | | Negative by both PCR and microscopy n(%) |
| Junju | 4910 | 790 (16.1) | 688 (14.0) | 22 (0.45) | | 3410 (69.45) |
| Ngerenya | 2406 | 5 (0.21) | 44 (1.83) | 0 (0) | | 2357(97.96) |
| Ganze | 1265 | 12 (0.95) | 62 (4.90) | 1(0.08) | | 1190(94.07) |
| Total | 8581 | 807 (9.40) | 794 (9.25) | 23 (0.26) | | 6957(81.07) |

**Supplementary Table 2.** Association between parasite prevalence by PCR and parasite prevalence by RDT at various grid sizes

| Site | Year | Parasite Prevalence | | 0.5x0.5 km^2^ Grid | | 1x1 km^2^ Grid | | 2x2 km^2^ Grid | |
| --- | --- | --- | --- | --- | --- | --- | --- | --- | --- |
|  |  | PCR (%) | RDT (%) | Correlation (CI) | P-value | Correlation (CI) | P-value | Correlation (CI) | P-value |
| Junju | Overall | 30.1 | 20.2 | 0.58 (0.53 - 0.63) | <0.001 | 0.66 (0.60 - 0.71) | <0.001 | 0.67 (0.60-0.76) | <0.001 |
|  | 2007 | 29.82 | 15.7 | 0.68 (0.46 - 0.82) | <0.001 | 0.69 (0.35 - 0.87) | <0.001 | 0.89 (0.54-0.98) | 0.002 |
|  | 2008 | 47.51 | 21.5 | 0.65 (0.42 - 0.81) | <0.001 | 0.53 (0.09 - 0.79) | 0.021 | 0.93 (0.69-0.99) | <0.001 |
|  | 2009 | 31.45 | 15.8 | 0.56 (0.30 - 0.75) | <0.001 | 0.69 (0.34 - 0.87) | 0.001 | 0.87 (0.49-0.97) | 0.002 |
|  | 2010 | 39.32 | 13.9 | 0.52 (0.36 - 0.64) | <0.001 | 0.49 (0.25 - 0.67) | <0.001 | 0.35 (-0.08-0.66) | 0.1045 |
|  | 2011 | 26.93 | 5.3 | 0.56 (0.43 - 0.67) | <0.001 | 0.73 (0.59 - 0.83) | <0.001 | 0.77 (0.55-0.89) | <0.001 |
|  | 2012 | 27.68 | 22.4 | 0.58 (0.45 - 0.69) | <0.001 | 0.66 (0.50 - 0.78) | <0.001 | 0.79 (0.57-0.90) | <0.001 |
|  | 2013 | 19.42 | 14.6 | 0.63 (0.51 - 0.73) | <0.001 | 0.79 (0.67 - 0.87) | <0.001 | 0.89 (0.77-0.95) | <0.001 |
|  | 2014 | 30.32 | 37.1 | 0.71 (0.61 - 0.79) | <0.001 | 0.88 (0.81 - 0.93) | <0.001 | 0.89 (0.77-0.95) | <0.001 |
|  | 2015 | 30.75 | 32.8 | 0.55 (0.29 - 0.74) | <0.001 | 0.53 (0.13 - 0.78) | 0.0135 | 0.86 (0.49-0.97) | 0.002 |
|  | 2016 | 23.51 | 28.9 | 0.54 (0.26 - 0.73) | <0.001 | 0.62 (0.23 - 0.84) | 0.005 | 0.91 (0.66 - 0.98) | <0.001 |
|  | Overall | 5.98 | 1.67 | 0.44 (0.33 - 0.54) | <0.001 | 0.47 (0.32 - 0.59) | <0.001 | 0.52 (0.33 - 0.67) | <0.001 |
| Ganze | 2012 | 8.18 | 2.99 | 0.56 (0.42 - 0.67) | <0.001 | 0.48 (0.28 - 0.64) | <0.001 | 0.59 (0.34 - 0.77) | <0.001 |
|  | 2013 | 3.94 | 0.44 | 0.23 (0.04 - 0.41) | 0.0170 | 0.44 (0.21 - 0.62) | <0.001 | 0.37 (0.03 - 0.63) | 0.0327 |
| Ngerenya | Overall | 2.04 | 0.38 | 0.40 (0.22 - 0.56) | <0.001 | 0.85 (0.78 - 0.90) | <0.001 | 0.37 (0.18 - 0.53) | <0.001 |

**Supplementary Table 3**: Association between distribution of malaria parasite prevalence detected by microscopy, PCR and RDT within 0.5x0.5 km^2^ grid size over time intervals.

|  |  | 0.5x0.5 km^2^ Grid | | | | | |
| --- | --- | --- | --- | --- | --- | --- | --- |
| Study Site | Interval between cluster (year) | Microscopy Analysis | | PCR Analysis | | RDT Analysis | |
|  |  | Correlation (95%CI) | P-value | Correlation (95%CI) | P-value | Correlation (95%CI) | P-value |
| Junju | 1 | 0.46 (0.40 - 0.52) | <0.001 | 0.40 (0.33-0.46) | <0.001 | 0.32 (0.25-0.39) | <0.001 |
|  | 2 | 0.46 (0.40 - 0.53) | <0.001 | 0.34 (0.27-0.42) | <0.001 | 0.34 (0.26-0.41) | <0.001 |
|  | 3 | 0.31 (0.22 - 0.39) | <0.001 | 0.28 (0.19-0.37) | <0.001 | 0.11 (0.02-0.21) | 0.0233 |
|  | 4 | 0.30 (0.20 - 0.40) | <0.001 | 0.30 (0.19-0.40) | <0.001 | 0.07 (-0.05-0.18) | 0.2465 |
|  | 5 | 0.31 (0.17 - 0.43) | <0.001 | 0.19 (0.05-0.33) | 0.009 | 0.05 (-0.09-0.19) | 0.4637 |
|  | 6 | 0.25 (0.09 - 0.40) | 0.0022 | 0.18 (0.02-0.34) | 0.0313 | 0.11 (-0.06-0.27) | 0.2092 |
|  | 7 | 0.19 (0.001 - 0.37) | 0.0494 | 0.17 (-0.02-0.35) | 0.0748 | 0.18 (-0.02-0.35) | 0.0719 |
|  | 8 | 0.28 (0.05 - 0.49) | 0.0207 | 0.11 (-0.13-0.34) | 0.3554 | 0.16 (-0.09-0.38) | 0.2038 |
|  | 9 | 0.27 (-0.08 - 0.57) | 0.122 | -0.04 (-0.38 - 0.30) | 0.8105 | -0.04 (-0.30 - 0.38) | 0.8253 |
| Ganze | 1 | -0.05 (-0.40-0.31) | 0.798 | 0.39 (0.04-0.65) | 0.0308 | - | - |

**Supplementary Table 4**: Association between distribution of malaria parasite prevalence detected by microscopy, PCR and RDT within 1x1 km^2^ grid size over time intervals.

|  |  | 1x1 km^2^ Grid | | | | | |
| --- | --- | --- | --- | --- | --- | --- | --- |
| Study Site | Interval between cluster (year) | Microscopy Analysis | | PCR Analysis | | RDT Analysis | |
|  |  | Correlation (95%CI) | P-value | Correlation (95%CI) | P-value | Correlation (95%CI) | P-value |
| Junju | 1 | 0.46 (0.37 - 0.54) | <0.001 | 0.41 (0.32-0.49) | <0.001 | 0.44 (0.35-0.52) | <0.001 |
|  | 2 | 0.49 (0.40 - 0.58) | <0.001 | 0.40 (0.29-0.49) | <0.001 | 0.44 (0.34-0.53) | <0.001 |
|  | 3 | 0.33 (0.21 - 0.45) | <0.001 | 0.34 (0.22-0.45) | <0.001 | 0.16 (0.03-0.29) | 0.0175 |
|  | 4 | 0.35 (0.21 - 0.48) | <0.001 | 0.38 (0.23-0.50) | <0.001 | 0.12 (-0.04-0.28) | 0.1394 |
|  | 5 | 0.27 (0.08 - 0.45) | 0.006 | 0.15 (-0.05-0.34) | 0.1403 | 0.03 (-0.17-0.22) | 0.794 |
|  | 6 | 0.29 (0.07 - 0.48) | 0.01 | 0.19 (-0.03-0.39) | 0.094 | 0.08 (-0.14-0.30) | 0.4575 |
|  | 7 | 0.20 (-0.05- 0.44) | 0.1142 | 0.19 (-0.07-0.42) | 0.1525 | 0.05 (-0.21-0.30) | 0.7078 |
|  | 8 | 0.23 (-0.09- 0.51) | 0.1616 | 0.07 (-0.25-0.38) | 0.6712 | 0.03 (-0.29-0.34) | 0.871 |
|  | 9 | 0.34 (-0.14- 0.69) | 0.1574 | 0.04 (-0.42-0.48) | 0.8789 | -0.20 (-0.60-0.28) | 0.4059 |
| Ganze | 1 | -0.05 (-0.40-0.31) | 0.798 | 0.39 (0.04-0.65) | 0.0308 | - | - |
